# Supplementary material for: SCHENGEN receptor module drives localized ROS production and lignification in plant roots
Source: EMBO J. 2020 Mar 18;39(9):e103894. doi: 10.15252/embj.2019103894 (PMC7196915; doi:10.15252/embj.2019103894)
Supplement: Supplementary file 1 — Appendix [file EMBJ-39-e103894-s001.pdf]

Appendix Fujita et al.

TABLE OF CONTENTS

Appendix Figure S1

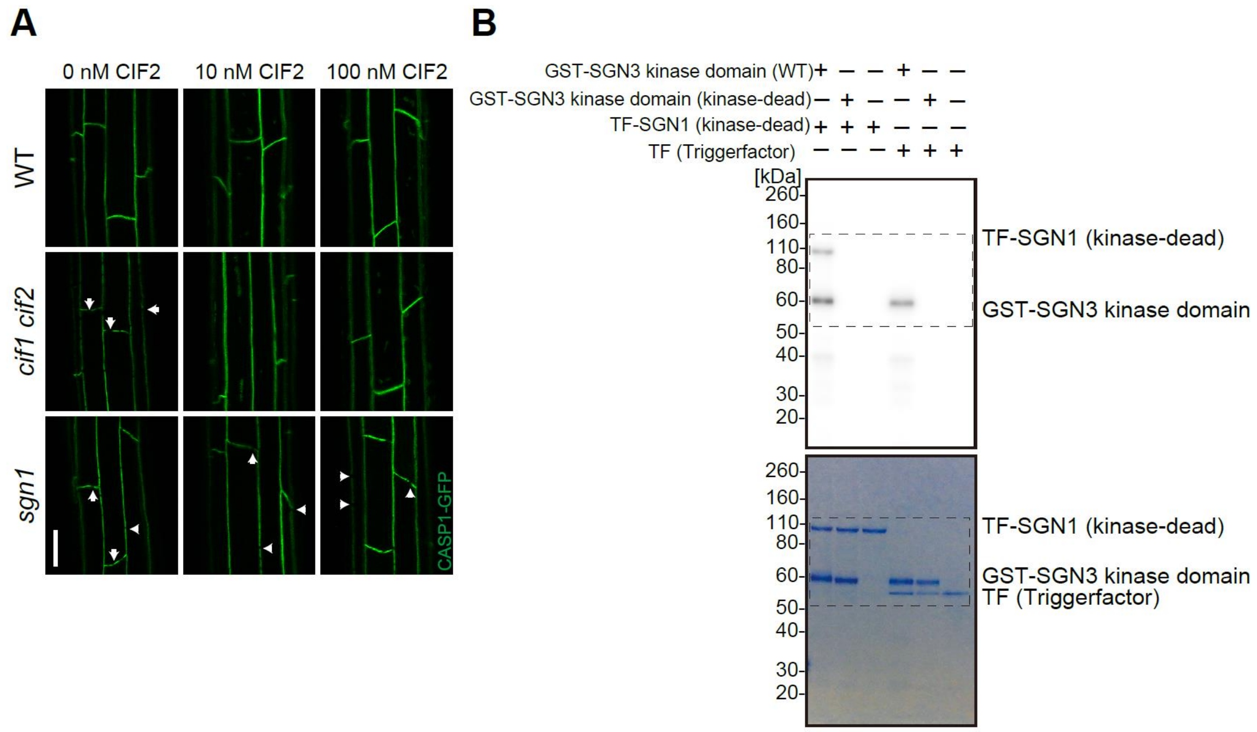

Appendix Figure S1 SGN1 acts as a transducer of CIF2 signaling and is phosphorylated by the SGN3 receptor

(A) Representative images of CASP1-GFP localization pattern after 5-day in the presence or absence of 10 or 100 nM CIF2. These pictures are part of the data used for quantification in Fig2A. Arrowheads highlight some of the discontinuities in the mutant CSDs. Scale bar = 20 µm.

(B) Full scan of the gels corresponding to Figure 2C. The regions in the dashed boxes are presented in Fig. 2C.
