# Supplementary material for: SCHENGEN receptor module drives localized ROS production and lignification in plant roots
Source: EMBO J. 2020 Mar 18;39(9):e103894. doi: 10.15252/embj.2019103894 (PMC7196915; doi:10.15252/embj.2019103894)
Supplement: Supplementary file 2 — Expanded View Figures PDF [file EMBJ-39-e103894-s002.pdf]

Expanded View Figures

Figure EV1. Apolar SGN1 leads to ectopic lignin accumulation in endodermal cells.

A PI penetration assay. Scoring of number of cells after the onset of cell elongation until PI signal is excluded from the inner side of the endodermis (10 roots in total were tested in each condition during two independent assays). In the box plot, boxes are showing ranges from the first to third quartiles, and the bold central lines display median. Upper and lower whiskers extend to the maximum or minimum values no further than 1.5 times IQR. Different letters indicate significant statistical differences ($P < 0.01$, one-way ANOVA and Tukey test).

B Localization of CASP1-mCherry, driven by CASP1 promoter, in *pCASP1::SGN1-Citrine* or *pCASP1::myrpalmSGN1-Citrine* transgenic lines. For each crossed line, more than 10 roots were observed and showed similar localization patterns. Scale bar = 10 μm.

C Localization patterns of SGN1 (WT, kinase dead (KD)), myrpalm-SGN1 (WT and KD), and lignin deposition patterns in each indicated transgenic line. Arrowheads indicate excess lignification. For this experiment, two independent lines were tested. From each transgenic line, 2 spots from 12 roots were observed and representative pictures are shown. Scale bars are 10 μm in SGN1-Cit, 5 μm in lignin and cell wall pictures, 20 μm in overview of lignin deposition.

D One-base pair insertion sites of *cif1-2* and *cif2-2*. Red letters indicate inserted bases in each locus.

E PI penetration phenotype of the *cif1 cif2* double mutant with or without 100 nM peptide treatment. Seedlings were germinated on the medium with or without peptides. At least five roots were observed in each condition. Asterisks indicate the stele. Scale bar = 40 μm.

F Whole root views of suberin deposition patterns in polar- or apolar-SGN1 transgenic lines. *esb1* (*enhanced suberin 1*) is shown as a representative oversuberized mutant. Scale bar = 500 μm.

G Quantification of the ratio of suberized zones and root lengths in each mutant or transgenic line. *esb1* is shown as a representative oversuberized mutant. In the box plot, boxes are showing ranges from the first to third quartiles, and the bold central lines display median. Upper and lower whiskers extend to the maximum or minimum values no further than 1.5 times IQR. Different letters are indicating statistically significant differences (n = 16–36 roots, $P < 0.01$, ANOVA and Tukey test).

© 2020 The Authors

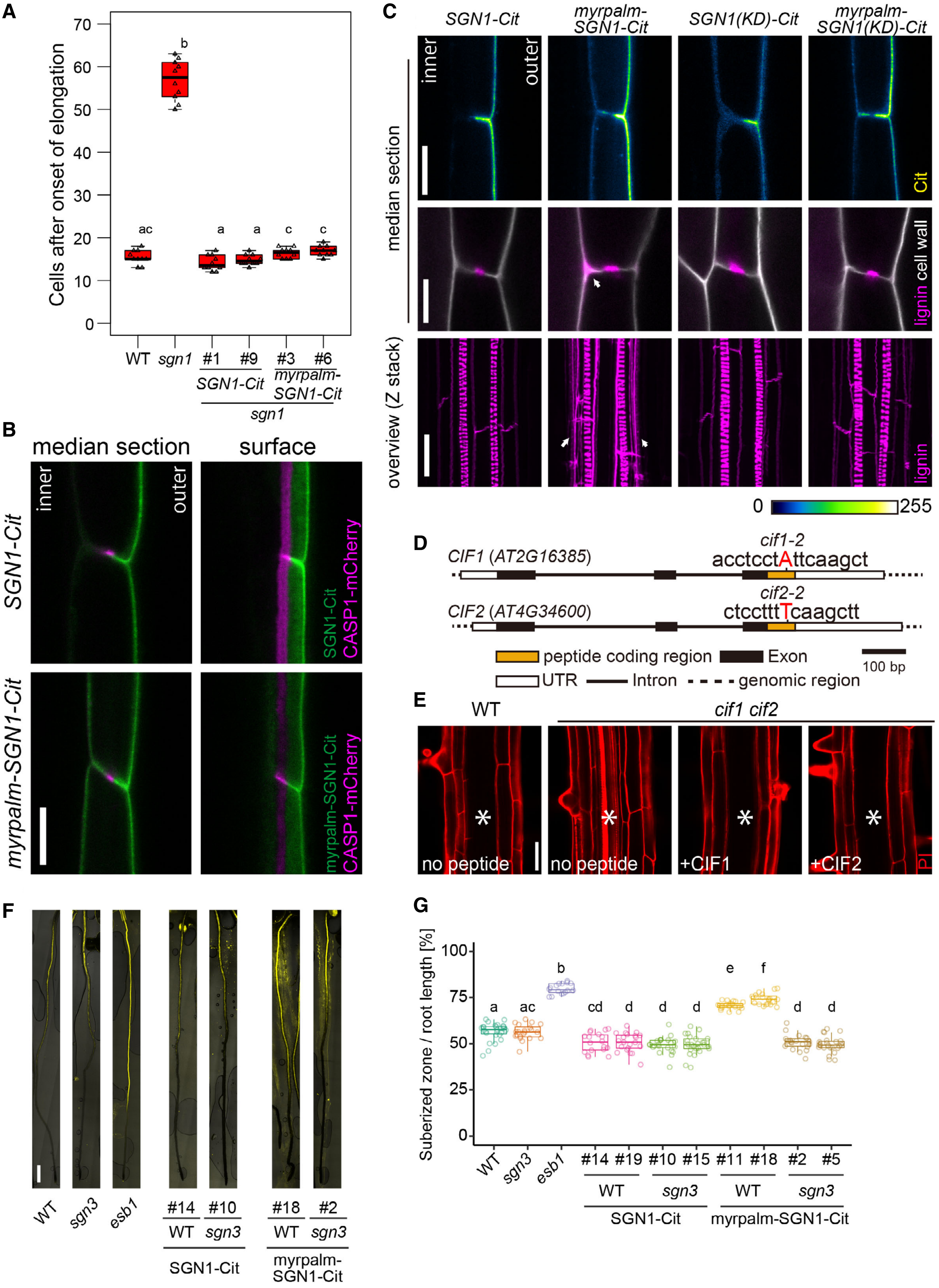

Figure EV1.

 © 2020 The Authors

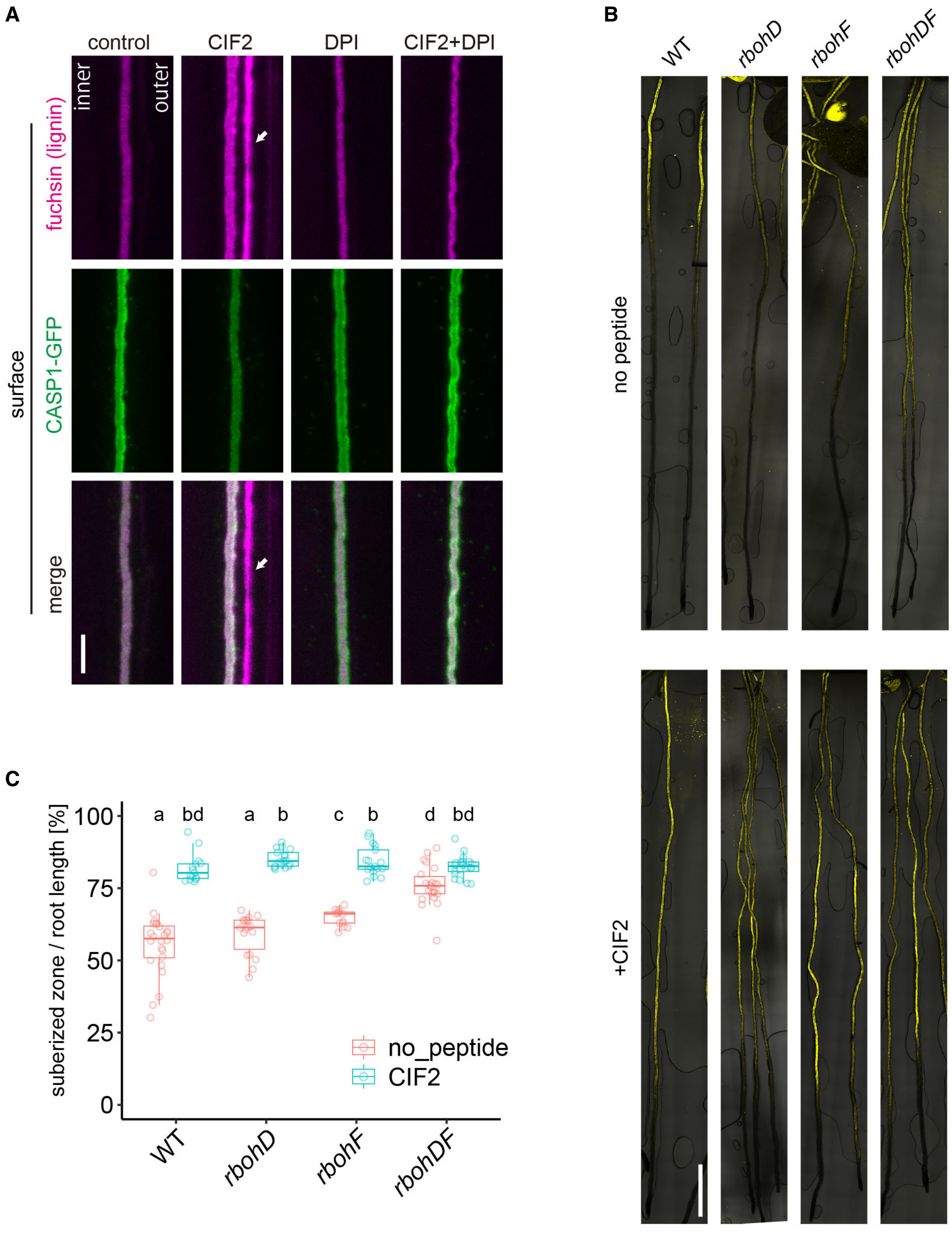
A
control
CIF2
DPI
CIF2+DPI
inner
outer
fuchsin (lignin)
CASP1-GFP
merge
surface
B
WT
rbohD
rbohF
rbohDF
no peptide
+CIF2
C
suberized zone / root length [%]
100
75
50
25
0
a
bd
a
b
c
b
d
bd
no_peptide
CIF2
WT
rbohD
rbohF
rbohDF

Figure EV2.

Figure EV2. Both RBOHD and F are required for CIF2-induced excess lignin accumulation.

A Co-treatment experiments with the NADPH oxidase inhibitor DPI (diphenyleneiodonium chloride) and CIF2. Pretreatment was done on medium with or without DPI for 30 min, and seedlings were then transferred to each medium (100 nM for CIF2 and 10 µM for DPI, respectively). The seedlings were incubated for 2 h in each condition. Arrowheads indicate excess lignification on the cortex-facing side. Representative pictures are shown from two independent experiments (2 spots from 5 roots in each condition for one experiment) with similar results. Scale bar = 5 µm.

B Whole root views of suberin deposition patterns in the *rbohD* and *rbohF* mutants with or without CIF2 treatment. Five-day-old seedlings were treated for 24 h with or without CIF2 and stained. Scale bar = 1 mm.

C Quantification of the ratio of suberized zones to root lengths in each mutant from (B) (n = 13–24 roots). In the box plot, boxes are showing ranges from the first to third quartiles, and the bold central lines display median. Upper and lower whiskers extend to the maximum or minimum values no further than 1.5 times IQR. Different letters indicate statistical significance (one-way ANOVA, Tukey's test).

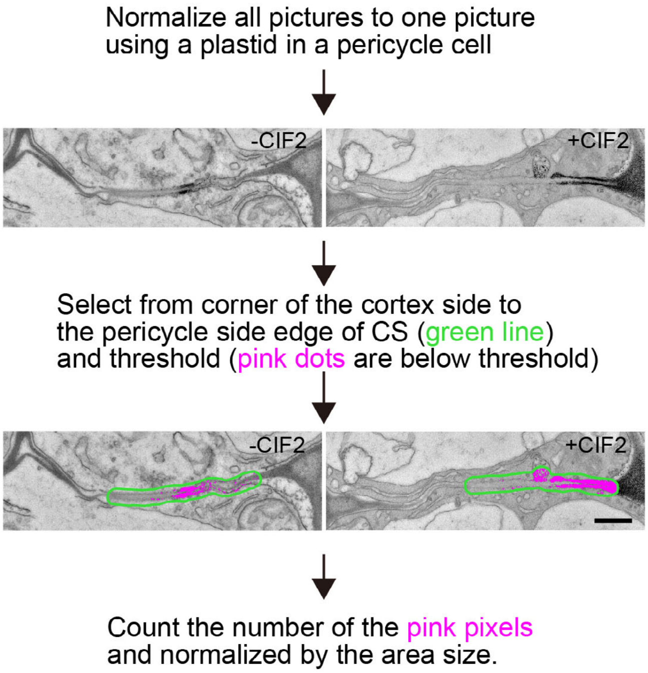

Figure EV3. ROS production is enhanced by SGN3/CIFs and requires RBOHD and F.

A schematic illustrating the protocol for pixel area quantification of ROS as measured by the cerium chloride method. Pictures were normalized to a picture of non-treated WT. Following the normalization, the area was chosen manually from the cortex side corner to the end of the CS at the pericycle side. Pixels below the threshold were marked as pink dots and counted. For more details, see the materials methods part. Note that the picture of WT (+CIF2) was reused from Fig 4D. Scale bar = 1000 nm.

 © 2020 The Authors

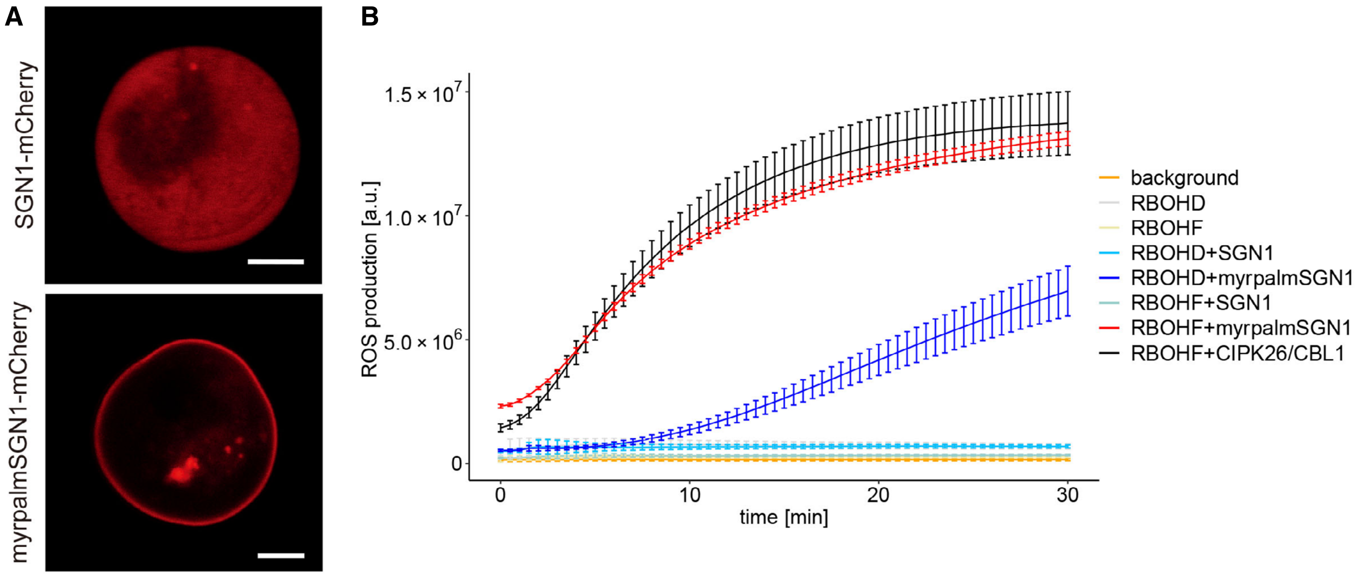

Figure EV4. SGN1 directly activates NADPH oxidases in a cellular context.

A Localization patterns of SGN1-mCherry of myrpalm-SGN1 in HEK293T cells. Myrpalm-SGN1 is efficiently recruited to the plasma membrane, while wild-type SGN-mCherry fusions remain in the cytoplasm. Scale bar = 5 µm.

B Independent HEK cell ROS production assay. The phosphatase inhibitor CalyculinA was added directly before the start of the measurements. Each data point represents the mean of six wells analyzed in parallel. Bars indicate SD.

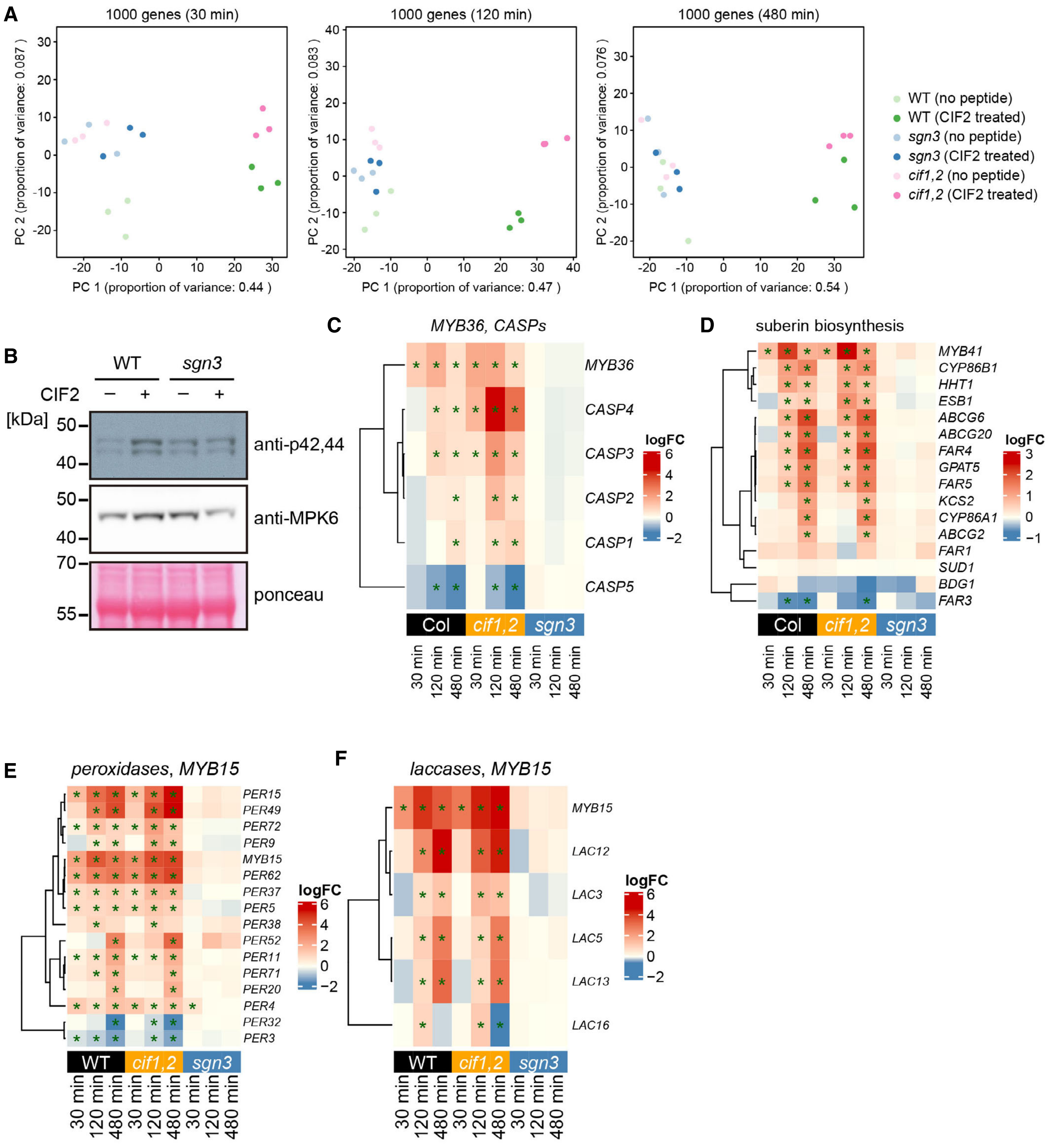

Figure EV5.

© 2020 The Authors

Figure EV5. CIF2 induces large-scale transcriptional changes to remodel cell walls.

A PCA of the most differentially expressed genes. The 1,000 most differentially expressed genes are clustered by condition at each time point for all replicates.
B Immunoblot with phospho-specific antibody against p42,44. Seedlings were treated with or without 1 μM CIF2 peptide for 15 min. Ponceau S-stained membranes and IB with anti-MPK6 antibody were shown as loading controls. This experiment was repeated three times with independent biological samples with the same result.
C Heatmaps of *MYB36* and *CASPs* expression fold changes with or without peptide treatment at the indicated time points. Asterisks indicate significant differentially regulated transcripts ($P \leq 0.05$) at each condition.
D Heatmaps of *MYB41* and suberin biosynthesis-related gene expression fold changes with or without the peptide treatment at the indicated time points. Asterisks indicate significant differentially regulated transcripts ($P \leq 0.05$) at each condition.
E Heatmaps of *MYB15* and *PEROXIDASES* expression fold changes with or without peptide treatment at the indicated time points. Asterisks indicate significant differentially regulated transcripts ($P \leq 0.05$) at each condition.
F Heatmaps of *MYB15* and *LACCASES* expression fold changes with or without the peptide treatment at the indicated time points. Asterisks indicate significant differentially regulated transcripts ($P \leq 0.05$) at each condition.

© 2020 The Authors
